# NIR-triggered thermosensitive polymer brush coating modified intraocular lens for smart prevention of posterior capsular opacification

**DOI:** 10.1186/s12951-023-02055-2

**Published:** 2023-09-07

**Authors:** Chen Qin, Shimin Wen, Fan Fei, Yuemei Han, Haiting Wang, Hao Chen, Quankui Lin

**Affiliations:** National Engineering Research Center of Ophthalmology and Optometry, School of Biomedical Engineering, School of Ophthalmology and Optometry, Eye Hospital, Wenzhou Medical University, Wenzhou, 325027 China

**Keywords:** Posterior capsule opacification, Drug-phototherapy, Thermosensitive coating, Controllable drug release, SI-RAFT

## Abstract

Posterior capsule opacification (PCO) is the most common complication after cataract surgery. Drug-eluting intraocular lens (IOLs) is a promising concept of PCO treatment in modern cataract surgery. However, the large dose of drugs in IOL leads to uncontrollable and unpredictable drug release, which inevitably brings risks of overtreatment and ocular toxicity. Herein, a low-power NIR-triggered thermosensitive IOL named IDG@P(NIPAM-*co*-AA)-IOL is proposed to improve security and prevent PCO by synergetic controlled drug therapy and simultaneous photo-therapy. Thermosensitive polymer brushes Poly(N-isopropylacrylamide-*co*-Acrylic acid) (P(NIPAM-*co*-AA)) is prepared on IOL via surface-initiated reversible addition-fragmentation chain transfer (SI-RAFT) polymerization. Then, Doxorubicin (DOX) and Indocyanine green (ICG) co-loaded Gelatin NPs (IDG NPs) are loaded in P(NIPAM-*co*-AA) by temperature control. The IDG NPs perform in suit photodynamic & photothermal therapy (PTT&PDT), and the produced heat also provides a trigger for controllable drug therapy with a cascade effect. Such functional IOL shows excellent synergistic drug-phototherapy effect and NIR-triggered drug release behavior. And there is no obvious PCO occurrence in IDG@P(NIPAM-*co*-AA) IOL under NIR irradiation compared with control group. This proposed IDG@P(NIPAM-*co*-AA)-IOL serves as a promising platform that combines phototherapy and drug-therapy to enhance the therapeutic potential and medication safety for future clinical application of PCO treatment.

## Introduction

Cataract, an opacification of native lens, is still the leading global causes of reversible blindness and visual impairment [1, 2]. According to the results of the 2020 epidemiological study, it has affected 15.2 million people those aged 50 years and older, and is estimated to get worse due to a global aging [3, 4]. The only effective treatment for cataract is phacoemulsification cataract surgery (PCS) [5]. It involves a tiny corneal incision and replaces the native lens with an intraocular lens (IOLs) to restore clear vision. Unfortunately, despite the high success rate of PCS, there have been adverse postoperative outcomes including posterior capsular opacification (PCO), intraocular inflammation, and infection [6]. Among them, PCO is more frequent especially in younger age, diabetes, and extracapsular cataract extraction. The results from a more than twenty years meta-analysis showed that incidence of PCO was 11.8% at 1 year, 20.7% at 3 years, and 28.4% at 5 years after surgery [7]. Although the surgical trauma is small, it can still induce a wound healing response, stimulate the proliferation and differentiation of the remaining LECs in lens capsule, and eventually cause visual impairment [8, 9]. Nd:YAG laser surgery can be safely addressed with PCO capsulotomy, while it is an extra treatment with its own set of potential complications such as transient increase of intraocular pressure, anterior uveitis, intraocular lenses pitting, cystoid macula edema, and retinal detachment [10–12]. Although materials and surgical techniques have improved over these years, the persistently high morbidity rates demonstrate the importance of finding new avenues to combat the underlying PCO.

The modified intraocular lens has been developed for many years as a drug delivery device, and it is a very promising concept in modern cataract surgery. This interest was sparked by the fact that the only widely used commercially available method to address potential PCO is the administration of eye drops postoperatively, while the majority of postoperative cataract patients show poor compliance and eye drops has low drug utilization [13]. Various drug-eluting IOLs have been designed to sustainably release drugs that reduce the risk of infection, suppress ocular inflammation, inhibit the proliferation of lens epithelial cells (LECs), and prevent the development of PCO following cataract surgery. For example, researchers have designed the sustained drug-eluting IOLs loaded with drugs like anti-inflammatory drug indomethacin [14] or bromfenac [15], anti-proliferative drug Cyclosporin A (CsA) [16] or Doxorubicin (DOX) [17], immunosuppressant rapamycin [18], infection prevention nanoparticles like zwitterionic silver nanoparticles [19–21] and so on. Nevertheless, due to the complex internal environment of the human eyes, this drug release is usually uncontrollable and unpredictable, which were had limitations in terms of ocular toxicity, excessive drug loading, and a short release time [22]. Compared with other organs, human eye is natural light-transmitting. External light can easily reach the lens through the pupil [23], like femtosecond laser refractive surgery [24] or fundus laser surgery [25]. To date, drug release platforms based on light response have been extensively explored and its stimulant reliability has also been recognized. Compared with other stimuli, light provides the advantages of noninvasiveness, simple remote controllability, and high spatiotemporal resolution [26].

At the same time, phototherapy has attracted increasing attention as a minimally invasive technique. It can convert NIR laser irradiation effectively into thermal energy or reactive oxygen species by activating photo conversion agents, which induce the death of cells through thermal ablation or oxidative stress damage [27, 28]. Thus, the IOL integrated photosensitizers or heat-generating graphene or metallic material have also become a promising candidate for PCO therapy. There were several nanoplatform-based IOLs with chlorin e6 (Ce6) loaded to eliminate the LECs by photodynamic therapy (PDT). Also, some IOLs were integrated with polydopamine [29], graphene oxide [30], Au nanoparticle [31] to killing cells via photothermal therapy (PTT). Despite recent advancements, challenges remain in the field of photo-therapy. Due to the stability of photosensitizers, the potential toxicity of metal or graphene materials, and temperature attenuation of the unilluminated lens part does not reach the desired, a higher laser power (> 1 W/cm^2^) and long period of time are often necessary for optimal outcomes, which might bring risks of light damage, especially for corneal or fundus cells. Integrating the PDT and PTT will overcome the inherent limitation of each other. However, considered the mismatch between a PDT agent and PTT agent at region, two different lasers are required, which leads to more complicated treatment process. Thus, fabricating a low power single laser induced synergetic controlled drug therapy and simultaneous photo-therapy (PDT&PTT) system for complication prevention is appropriate and meaningful.

Indocyanine green (ICG) has been approved by Food and Drug Administration (FDA) as a medical diagnostic, especially in ophthalmic surgery. Its absorption peak in the near-infrared spectrum is around 800 nm, and this infrared frequency can penetrate deep into tissues, which makes ICG not only applicable for PTT and PDT but also for near-infrared fluorescence imaging, respectively [32]. However, due to degradation caused by the saturation of the double bonds in the conjugated chain, ICG in aqueous solution has an obvious decrease in absorption. This poor stability of ICG in water limits its use [33]. To improve the stability of ICG in aqueous condition, the most common strategy is to load it into nanocarriers, such as mesoporous Silica Nanoparticles [34] and Carbon nanoparticles [35].

Gelatin, a natural proteinaceous biomacromolecule, is also a safe material approved by FDA [36]. It is isolated from collagen found in animal connective tissues such as skin, bone, and tendon tissue. It has several advantages, including low cost, biocompatibility, biodegradability, and non-immunogenicity [37, 38]. It is a polymorph with both positive and negative ionic groups. This unique structure and properties constitute its success as a carrier system that can be applied to a wide variety of drug molecules. These characteristics make it an ideal nanocarrier as a nano platform for phototherapy and drug-therapy treatments.

Poly(N-isopropylacrylamide) (PNIPAM) is a representative thermos-responsive polymer. Due to its temperature sensitivity, PNIPAM polymers have promising applications in drug release, immobilized enzymes, material separation, immunoassays, etc [39]. It has a lower critical solution temperature (LCST) of around 32 ℃, which is between the temperature of the human body 37 ℃ and the room temperature of 25 ℃. This property gives him an advantage when applied to the human body [40]. Due to the role of hydrogen bonds, the PNIPAM will stretch when the temperature is below the T_LCST_, whereas it will collapse when the temperature is above the T_LCST_. Thus, when used in the human body, it will quickly gather, which limits its application. To change its T_LCST_, researchers usually add hydrophilic or hydrophobic monomers during the polymerization [41].

**Scheme 1.**
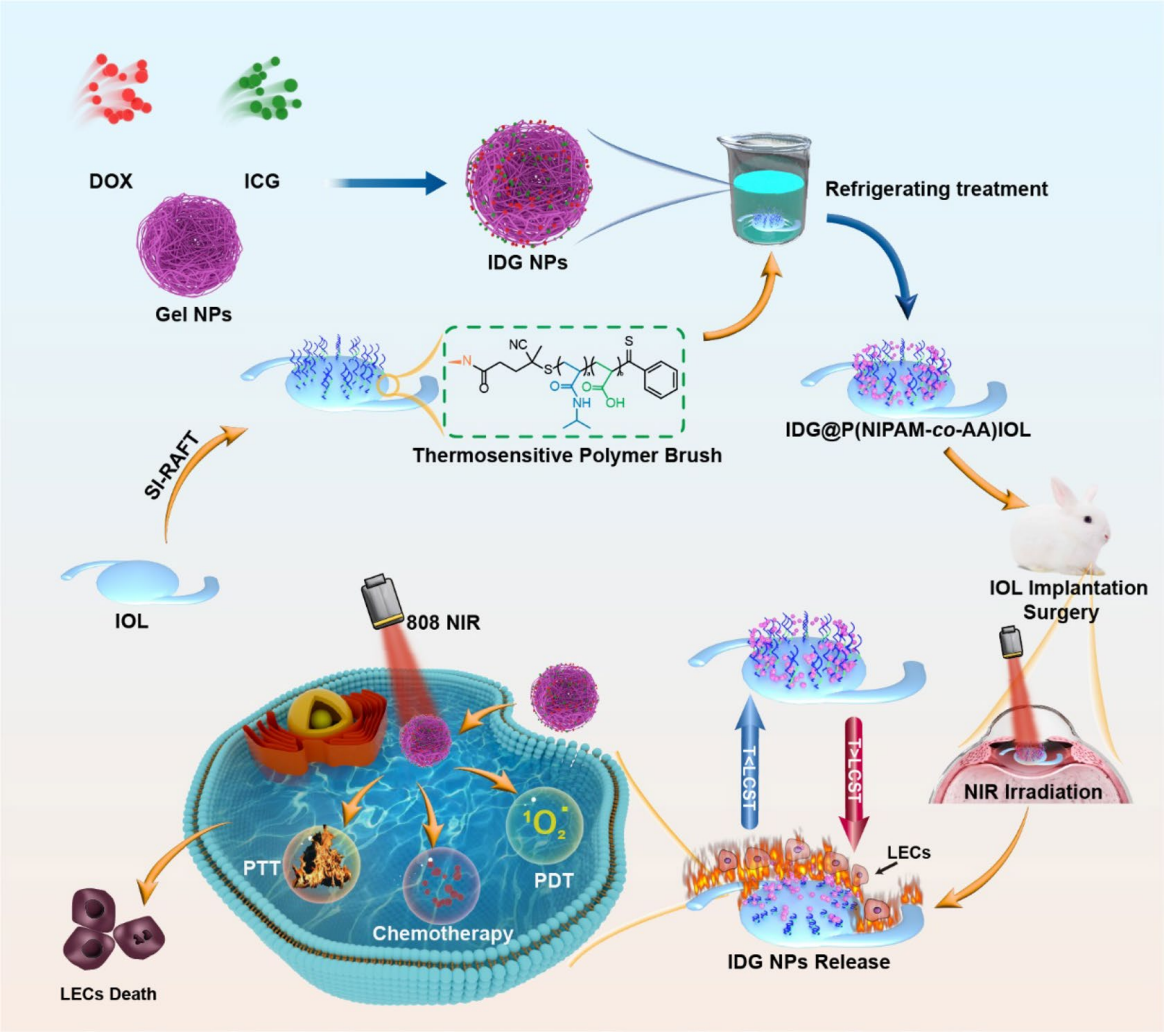
Schematic illustration of the fabrication of IDG@P(NIPAM-*co*-AA)-IOL and its application for synergistic phototherapy and controllable drug-therapy on PCO prevention

Herein, a low power NIR triggered IDG@P(NIPAM-*co*-AA)-IOL was designed and constructed by a thermosensitive P(NIPAM-*co*-AA) polymer brush coating loaded with dual-drug nanoparticle ICG&DOX@Gel NPs (IDG NPs) for both efficient and controllable synergistic drug-therapy and phototherapy to prevent PCO and improve security (Scheme 1). we prepare thermosensitive polymer brushes coating P(NIPAM-*co*-AA) by copolymerization with hydrophilic monomer acrylic acid (AA) on IOL surface via surface-initiated reversible addition-fragmentation chain transfer (SI-RAFT) polymerization, and successfully adjust the T_LCST_ to about 40 °C. The photoconversion IDG NPs was loading within the thermosensitive P(NIPAM-*co*-AA) polymer brushes. With the role of dual-stabilizing effect from Gel NPs and thermosensitive coating P(NIPAM-*co*-AA), this system avoided premature release at physiological temperature, which enhanced the drug application safety. When under low-power NIR irradiation, it performs not only both PDT and PTT effect, but also the quick heats up of coating triggers the collapse of P(NIPAM-*co*-AA) brushes and leads to the controllable drug (DOX) release and transparency decrease. Thus, this thermosensitive IDG@P(NIPAM-*co*-AA)-IOL can safely and efficiently inhibit LECs proliferation via controllable drug release and in-situ simultaneous PDT&PTT, which proposing a smart strategy for PCO prevention.

## Experiment section

### Materials

Gelatin (type A from porcine skin, gel strength > 250 g Bloom), Cell Counting Kit-8 (CCK-8), Calcein AM cell-permeant dye, and propidium iodide (PI), 2′,7′-dichlorofluorescein diacetate (DCFH-DA), 3,3′-dioctadecyloxacarbocyanine perchlorate (DIO), 2-(4-Amidinophenyl)-6-indolecarbamidine dihydrochloride (DAPI) were purchased from Beyotime Biotechnology (Shanghai, China). Phosphate-buffered saline (PBS) was purchased from Boster Biological Technology (Pleasanton, CA, USA). Doxorubicin hydrochloride (DOX·HCl) was purchased from Meilun Bio (Dalian, China). Indocyanine Green (ICG) and MES Buffered Solution (0.05 M, pH6.7) were purchased from Aladdin (Shanghai, China). Fetal bovine serum (FBS) and trypsin-ethylenediaminetetraacetic acid solution, penicillin–streptomycin solution, and Dulbecco’s modified Eagle’s medium/nutrient mixture F-12 (DMEM/F-12; 1:1 ratio) were purchased from Thermo Fisher Scientific (Carlsbad, CA, USA). Acrylic acid (AA), Polyethylenimine (PEI, branched, 25,000 Mw), N-Isopropylacrylamide (NIPAM), 4-Cyano-4-(phenylcarbonothioylthio)pentanoic acid (CTA agent), 4,4′-Azobis(4-cyanovaleric acid) (ABCVA, initiator agent), 1,3-diphenylisobenzonfuran (DPBF), Glutaraldehyde solution, N-(3-(dimethylamino) propyl)-N′-ethyl carbodiimide hydrochloride (EDC), N-Hydroxysuccinimide (NHS), Sodium dodecyl sulfate (SDS) and Acetylcysteine (NAC) were purchased from Sigma-Aldrich Co (Shanghai, China). Glycine was purchased from Macklin(Shanghai, China). Intraocular lens (IOL, hydrophobic acrylate) was purchased from Suzhou 66 Vision Tech Co., Ltd). All chemicals were of analytical grade and used following the manufacturers’ instructions.

### Preparation of Gel NPs and IDG NPs

The gelatin nanoparticles (Gel NPs) were prepared through two_step “double-desolvation” method according to the previous publications with minor improvement [29]. In brief, gelatin (625 mg) was dissolved into 12.5 mL deionized (DI) water at 50℃. Seal the solution carefully using parafilm and continuously stirring for 30 min. Next, 12.5 mL cold acetone was added and left for 60 min at room temperature. The supernatant that contains low molecular weight gelatin was discarded. Next, the remaining solution was dissolved into 12.5 mL deionized water at 50 ℃ and adjusted pH 2.5 through the 5 M HCl. Next, acetone was added dropwise (2 mL/min, 37 mL) into the solution under vigorously stirring (1650 rpm). The endpoint is the formation of a thick white-colored colloidal dispersion. To crosslink the gelatin nanoparticles, 82.5 μL glutaraldehyde (25% w/v) was slowly added into the solution under constant stirring overnight and the crosslinking reaction was stopped by equal volume of glycine solution (0.1 M) dropwise adding. Then, the mixed solution was washed by centrifugation and redissolution in DI water three times and thus the gelatin nanoparticles (Gel NPs) were obtained.

To prepare the ICG&DOX@Gel NPs (IDG NPs), 2 mL of Gel NPs solution, 250 μL of DOX solution (1 mg/mL), 250 μL of ICG solution (1 mg/mL), and 250 μL DI water were mixed sonication at least 30 min. Eventually, the solution was centrifuged 3 times to remove free ICG and DOX, the precipitate was redissolved in 2 mL DI water and stored at 4 ℃. For a comparison, mono drug-loaded gelatin nanoparticles were also prepared with the same method. The DOX@Gel NPs and ICG@Gel NPs were named as DG and IG, respectively.

### Characterizations of Gel NPs and IDG NPs

Dynamic light scattering (DLS) analysis involving particle size and zeta potential measurements was carried out using a DLS system (Malvern Instrument Ltd., Malvern, UK). High-resolution microscopy was performed using a transmission electron microscope (TEM, Thermo Fisher Talos F200S, US). The Ultraviolet–visible (UV–vis) absorption of nanoparticles is determined by UV–vis spectrophotometer (UV-1780, Shimadzu, Japan). The fluorescence spectra of nanoparticles are determined by a multi-functional ultraviolet enzyme labeling instrument (SpectraMax 190, USA).

During the process of IDG NPs preparation, the supernatant after centrifugation is used to estimate the loading capacity of ICG and DOX in IDG NPs. UV–vis spectrophotometer was used to quantitate the amount of free ICG or free DOX by determining the UV–vis absorption peak of ICG at 780 nm or DOX at 490 nm. Finally, the drug loading efficiency (LE) and encapsulation efficiency (EE) were calculated by the results of UV–vis.

### Synthesis of thermal responsive P(NIPAM-*co*-AA) brush on IOLs

The thermal responsive P(NIPAM-*co*-AA) brush coating was synthesized by SI-RAFT polymerization. First, clean IOLs were immersed in PEI solution (3 mg/mL) for 6 h for surface amination. 14 mg CTA agent, 22 mg NHS, and 38 mg EDC were mixed in 10 mL MES Buffer solution under continuous stirring for 30 min to activate the carboxyl in CTA agent, followed with the aminated IOLs adding. The mixture was continuously stirred at room temperature for 4 h and CTA-IOLs were obtained. NIPAM (565.8 mg), AA (288 mg), ABCVA (5.6 mg), and CTA-IOLs were mixed in 20 mL DI water, degased by nitrogen bubbling for 1 h and reacted in an oil bath at 60℃ for 8 h. Finally, the obtained P(NIPAM-*co*-AA)-IOLs were washed with DI water.

### Preparation and characterization of IDG@P(NIPAM-*co*-AA)-IOLs

The P(NIPAM-*co*-AA)-IOLs were firstly soaked in 60 °C DI water for 30 min, then immersed in 4 °C IDG solution for 2 days. After washing with DI water 3 times, the obtained IDG@P(NIPAM-*co*-AA)-IOLs were dried by nitrogen flow and preserved in dark. Also, the P(NIPAM-*co*-AA) merely loaded with DG NPs or IG NPs were also prepared with the same method, which was abbreviated as DG@P(NIPAM-co-AA) and IG@P(NIPAM-*co*-AA).

The IDG@P(NIPAM-*co*-AA) coating was analyzed by X-ray photoelectron spectroscopy (XPS) (Thermo Scientific Escalab 250Xi, Netherlands). Water contact angle (WCA) measurement was also performed at different temperatures. The optical properties were detected by UV–vis spectrophotometer and contrast sensitivity detection. For characterization, the IDG@P(NIPAM-*co*-AA) brush coating were simultaneously prepared on the surface of other materials according to the above method.

### Photothermal effect of IDG@P(NIPAM-*co*-AA)-IOLs

In order to investigate the vitro photothermal performance, the P(NIPAM-*co*-AA) brush coated materials were pre-immersed in the solution of PBS, Gel NPs, DOX@Gel NPs (DG), ICG@Gel NPs (IG), and IDG NPs for 3 days, respectively. Then, different nanoparticles-loaded materials were placed on the suspended scaffold, following irradiation with 808 nm laser for 300 s. The real-time temperature increasion in each group was recorded every 10 s by using an IR thermal camera (225RD-L39C, Fortic). The IR thermal images were then quantified by AnalyzIR software. To investigate the NIR laser power-dependent PTT efficiency, IDG@P(NIPAM-*co*-AA)-IOLs (immersion concentration: 50 µg/mL) were irradiated under NIR laser of different powers (0.2, 0.4, 0.6, 0.8, and 1 W/cm^2^) and recorded the temperature every 20 s. Besides, the IDG concentration-dependent PTT efficiency was also studied by the similar methods. The P(NIPAM-*co*-AA)-IOLs were immersed in different concentrations of IDG solution (20, 50, 100, 150, 200 µg/mL) and followed with 808 nm laser (0.38 W/cm^2^) for 300 s. Meanwhile, the PTT stability of the IDG@P(NIPAM-*co*-AA)-IOLs under five consecutive laser on/off cycles was also evaluated under similar conditions.

### Photodynamic effect of IDG@P(NIPAM-*co*-AA)-IOL

DPBF was used to study the vitro ROS generation from IOLs by measuring the quenching UV–vis absorption at 410 nm. The test was operated by immersing the IDG@P(NIPAM-*co*-AA)-IOLs in DPBF solution (10 µg/mL) with or without irradiation at room temperature (about 25 °C). The absorption of DPBF with IDG@P(NIPAM-*co*-AA)-IOLs (marked as coating) was recorded every 30 s from 0 to 120 s under irradiation (808 nm, 0.38 W/cm^2^), and every 5 min from 0 to 15 min without irradiation. As a control, the absorption of DPBF solution with unmodified IOLs was recorded every 3 min from 0 to 6 min under irradiation (808 nm, 0.38 W/cm^2^).

### Measurement of photothermal release of IDG NPs

The photothermal release studies were performed by detecting the concentration of IDG at 37 °C. In detail, the IDG@P(NIPAM-*co*-AA)-IOL was immersed in a cuvette containing 2 mL PBS. The cuvette was kept in a constant temperature shaking box (37 °C 100 rpm) for 24 h. After specific time intervals, the IOLs were irradiated by NIR laser (808 nm, 0.38 W/cm^2^) for about 3 min. Afterward, the solution was gently mixed, followed by using UV–vis spectrophotometer to quantitate the amount of photothermally released IDG NPs. As a control, the IDG@P(NIPAM-*co*-AA)-IOL in 2 mL PBS cuvette without NIR irradiation was used.

### Cell experiments

Human lens epithelial cells (HLECs) were bought from the Chinese Academy of Sciences Cell Bank (Shanghai, China), and were kept at 37 °C in a 5% CO_2_ humidified incubator. The DMEM media supplemented with 10% FBS was used as the culture media. All materials used for cell experiments are pre-sterilized by UV light for 30 min. Each cell culture experiment had at least three technical and three biological repeats.

### Intracellular ROS detection

For convenience, the IOLs was replaced with intraocular lens material (polyethylene terephthalate, PET), and small discs (96-well size) with the coatings containing different compositions on the surface were prepared as described above. HLECs were co-incubated with these small discs on 96-well plate (5000 cells per well) at 37 ℃ with 5% CO_2_ for 24 h. Afterward, the cells were treated with or without NIR irradiation (808 nm, 0.38 W/cm^2^, 5 min). Then DCFH-DA was added at a concentration of 10 µM, and the intracellular ROS generation was observed using a fluorescence microscope (Leica DMi8, Germany).

### Synergistic inhibition of drug-phototherapy

The cellular cytotoxicity induced by synergistic drug-phototherapy (PDT/PTT) was detected by CCK-8 assay. Typically, pre-sterilized discs of P(NIPAM-*co*-AA) brush, Gel@P(NIPAM-*co*-AA), DG@P(NIPAM-*co*-AA), IG@P(NIPAM-*co*-AA) and IDG@P(NIPAM-*co*-AA) were placed on the bottom side of a 96-well plate. Then, the HLECs were seeded at a density of 5 × 10^3^/well and incubated at 37 °C for 8 h. To induce synergistic drug-phototherapy, each well was then irradiated with NIR irradiation (808 nm, 0.38 W/cm^2^, 5 min) and incubated for 24 h. For comparison, the wells without illumination were also studied. Next, the CCK-8 agent was added and incubated for another 2 h. The absorbance at 450 nm for each well was recorded by Microplate Reader (SpectraMax 190, USA) to determine the cell viability.

Likewise, vitro synergistic properties of drug-phototherapy was further investigated through the co-staining with Calcein-AM and PI. Briefly, HLECs were incubated with each of the above materials in culture plates, irradiating each well with a NIR-laser for 5 min. Then co-stained with Calcein-AM and PI for 20 min. Cells were then washed with PBS and photographed under an inverted fluorescence microscope (Leica DMi8, Germany). Importantly, whether different coating discs under dark conditions or illumination, the no-coating material disc was used as a control. All conditions in the control and experimental groups remained unchanged except for the above.

### Cell uptake

HLECs were incubated on both blank 24-well cell slide and IDG@P(NIPAM-*co*-AA) cell slide. Briefly, 7.5 × 10^4^ cells in DMEM were plated on the above slides. After 4 h incubation, specific well was irradiated with NIR irradiation (808 nm, 0.38 W/cm^2^, 5 min). For another 2 h, cell membranes were stained with DIO dye (green), and the nuclei were stained with DAPI (blue), and finally imaged under Confocal microscopy (CLSM, Zeiss LSM 880, Germany).

### In vivo efficiency of photothermal therapy against PCO

The right eye of 2–3 month old New Zealand white rabbits was selected to establish the animal model after cataract surgery by phacoemulsification combined with intraocular lens implantation. The study was approved by the local Experimental Animal Committee. All rabbit eyes were routinely examined with a slit lamp before operation to rule out congenital intraocular lesions. Levofloxacin eye drops were routinely used three days before surgery. At the same time, IDG@P(NIPAM-*co*-AA)-IOLs were sterilized with ethylene oxide. All IOLs were successfully implanted into the lens capsule. All the rabbits were randomly divided into control group (Blank IOLs), light or non-light group (IDG@P(NIPAM-*co*-AA)-IOLs). There were 4 rabbits in each group, and all the operations were performed by Dr. Han.

Postoperative routine treatment included levofloxacin eye drops, tobramycin dexamethasone ointment and atropine eye drops. After surgery, the 808 nm light was given at a determined time (1d, 3d, 7d, 14d) for 5 min after operation. The acute ocular inflammatory reaction was examined with a slit lamp on the 1d, 3d, and 7d after operation, and the clinical color photos were taken with a digital camera attached to the slit lamp microscope at a certain time (7d, 14d, 21d, 28d). The posterior capsular opacification was obvious two weeks after operation. 28 days later, the animals were killed humanely and the eyes were examined for histological examination to determine the preventive effect of PCO.

### Statistical analysis

All data were expressed as mean ± SD (standard deviation) from at least three independent experiments. Statistically significant, one-way ANOVA analysis was used; p < 0.05 (*), p < 0.01 (**), or p < 0.001(***) are marked in the figures.

## Results and discussion

### Preparation and characterization of IDG NPs

**Fig. 1:**
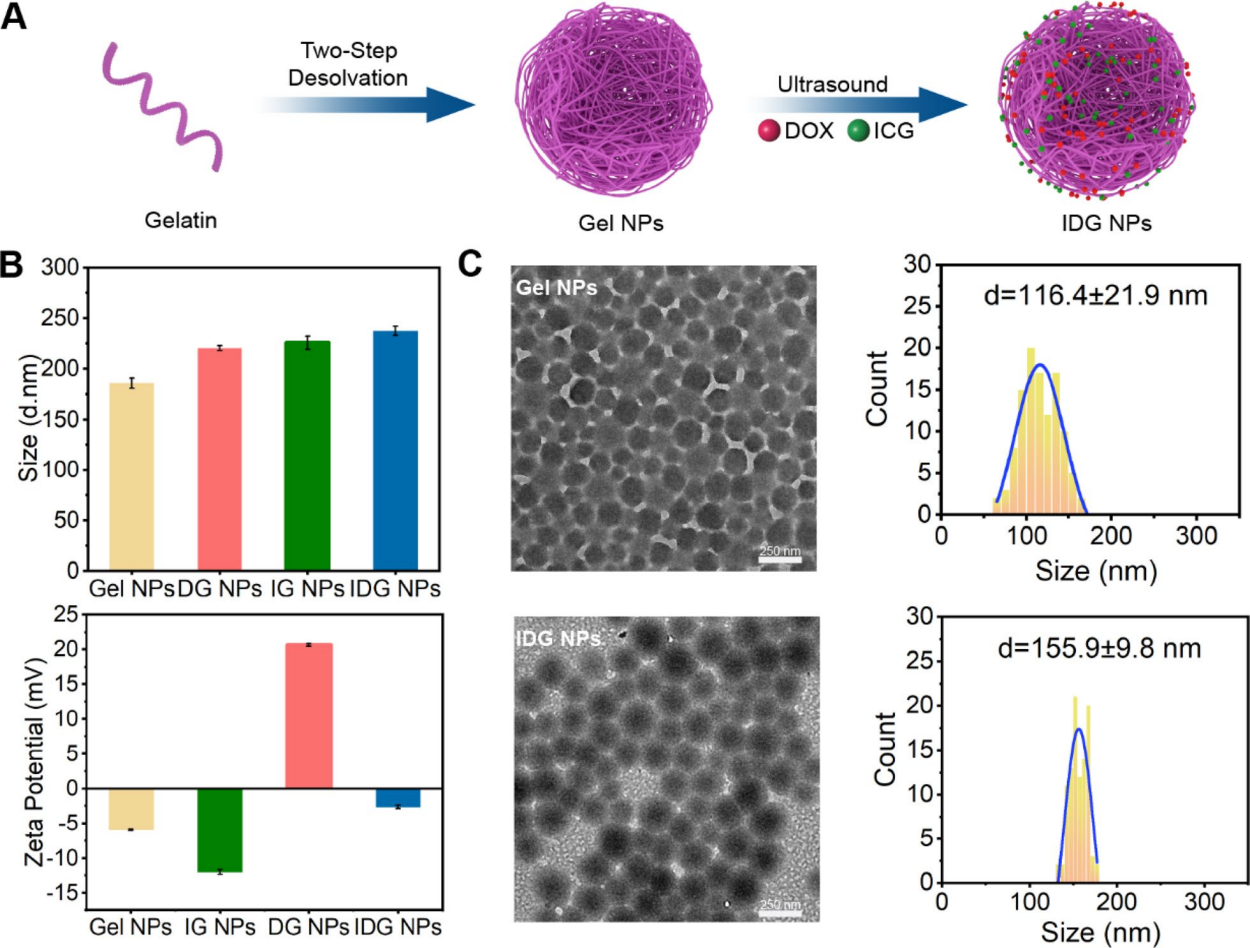
Fabrication and characterization of IDG NPs. **A** Schematic illustration on the fabrication of IDG NPs. **B** The size and zeta potential during the fabrication of Gel NPs, DG NPs, IG NPs, IDG NPs. **C** Representative TEM images of IDG NPs and the corresponding frequency distribution map

IDG NPs were prepared of gelatin NPs (Gel NPs) loaded with ICG and DOX. As shown in Fig. 1A, Gel NPs were first prepared by a modified two-step desolvation method. ICG and DOX were then loaded into the Gel NPs under sonication to obtain the IDG NPs. Noticeably, the addition of an equal volume of glycine solution was necessary. This step blocked the unreacted aldehyde groups of glutaraldehyde to stop the cross-linking reaction, making sure the synthesized Gel NPs was uniform in particle size. TEM image and DLS analysis revealed that Gel NPs had a spherical shape and good dispersibility with a diameter in the dehydrated state (DD) of 116.4 ± 21.9 nm and a hydrodynamic diameter (HD) of 185.9 ± 4.9 nm, and the polydispersity index (PDI) was 0.231 ± 0.035. The obtained IDG NPs also showed a spherical morphology, and the DD and HD were 155.9 ± 9.8 nm and 237.4 ± 4.59 nm, respectively, the PDI was 0.130 ± 0.024 (Fig. 1B, C). As compared with Gel NPs and DOX@Gel NPs (DG NPs) and ICG@Gel NPs (IG NPs), the IDG NPs exhibited a bigger size, which expressing the fact that both DOX and ICG could be encapsulated in the Gel NPs by sonication for 30 min (Fig. 1B). Zeta-potential measurements indicated that Gel NPs had a zeta potential of -5.99 mV. After loading with ICG and DOX, the whole zeta-potential became -2.68 mV, which was attributed to the positive charges carried by DOX.

**Fig. 2:**
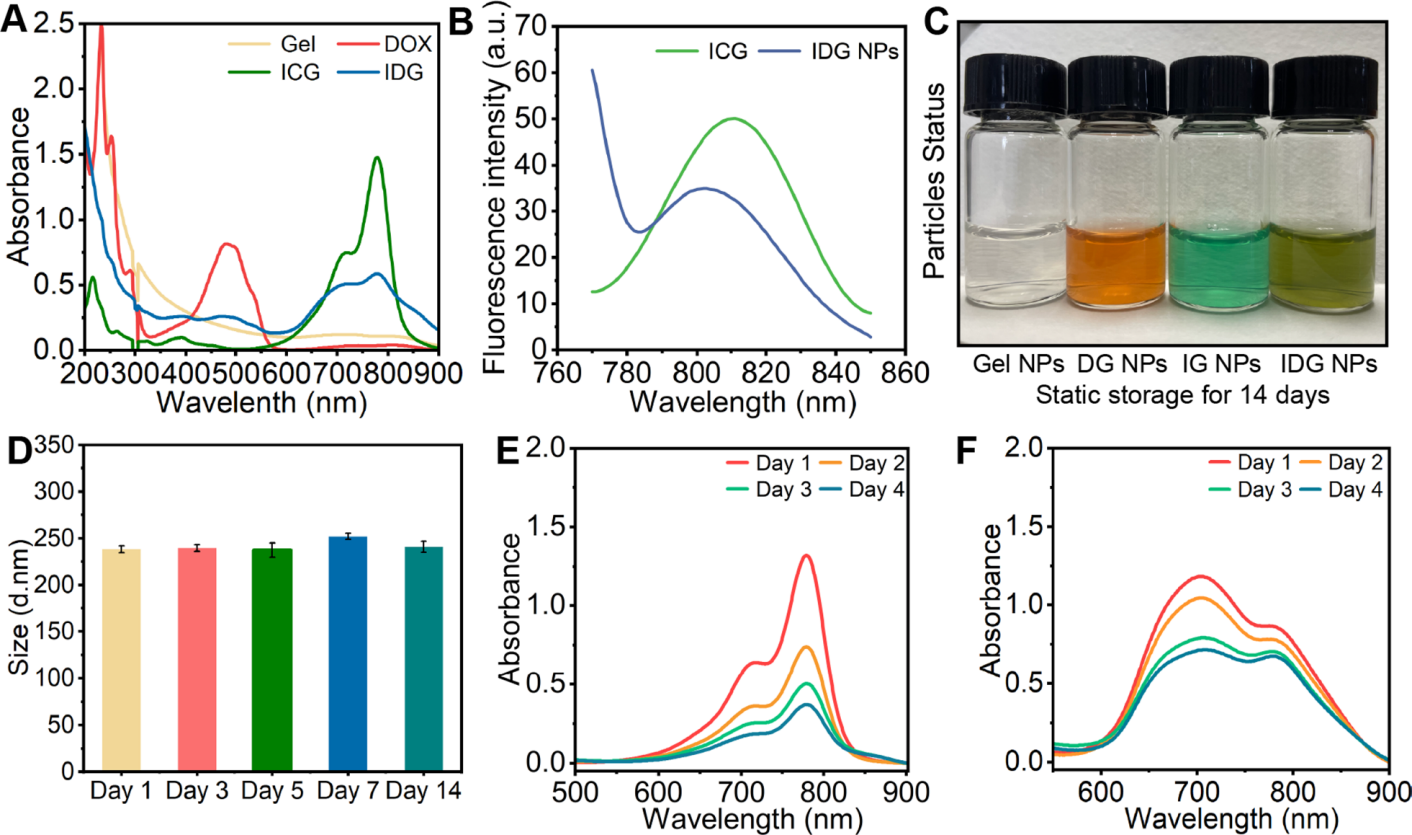
IDG characterization and stability. **A** UV − vis spectrum of homogeneous solutions Gel NPs, free DOX, free ICG, and IDG NPs that uniformly dispersed in DI water. **B** The characteristic fluorescence spectrum of free ICG and IDG NPs. **C** Status of four nanoparticles after storing for 14 days. **D** Changes of hydrodynamic diameter of IDG NPs during 14 days. **E**, **F** Absorption spectra changes of free ICG (**E**) and IDG (**F**) in aqueous solution during 4 days

In Fig. 2A and B, both ICG and IDG NPs exhibited similar NIR absorption and FL emission. In detail, the characteristic absorption peaks of ICG (780 nm) and DOX (480 nm) could be found in UV–vis spectrum of IDG NPs, which confirmed the successful drug loading into the IDG NPs. Also, comparing with free ICG, IDG NPs exhibit a subtle blue shift in fluorescence spectrum from 808 to 802 nm. This change was due to the interference of DOX and Gel NPs. While the fluorescence spectroscopy between ICG and IDG NPs were roughly similar around 805 nm, which means that IDG nanoparticles could still be excited by 808 nm NIR-laser (Fig. 2B). Here, the drug-loading contents of ICG and DOX in IDG NPs were 8.5% and 14%, and encapsulation efficiencies were determined to be 15.6% and 22%, respectively.

Besides, the long period stability was also taken into consideration. Four kinds of nanoparticles obtained from the preparation were placed in a 4 °C refrigerator for 14 days. As shown in Fig. 2C, Gel, IG, DG and IDG NPs all showed good dispersion properties without visible aggregated particles. In order to further evaluate the stability of IDG NPs, its particle size was measured at predetermined time during 14 days. As a results, even with dual-drug loading, IDG NPs also keep a relatively stable particle size around 235 nm (Fig. 2D). All above results demonstrated that IDG NPs was successfully prepared and showed long period uniform and stable.

Considering the poor aqueous stability of ICG, the absorption spectra of ICG and IDG in aqueous were measured at predetermined times after storing in a constant temperature shaker box at 37℃ with 100 rpm for 4 days. As a results, almost 72% absorption at 780 nm of ICG lost over a period of 4 days (Fig. 2E). While, the absorption of IDG still remain 78% of intensity after 4 days (Fig. 2F). Obviously, when ICG was encapsulated into Gel NPs, its stability improved, which protect the double bonds of ICG from saturation in solution.

### Preparation and characterization of IDG@P(NIPAM-*co*-AA)

**Fig. 3:**
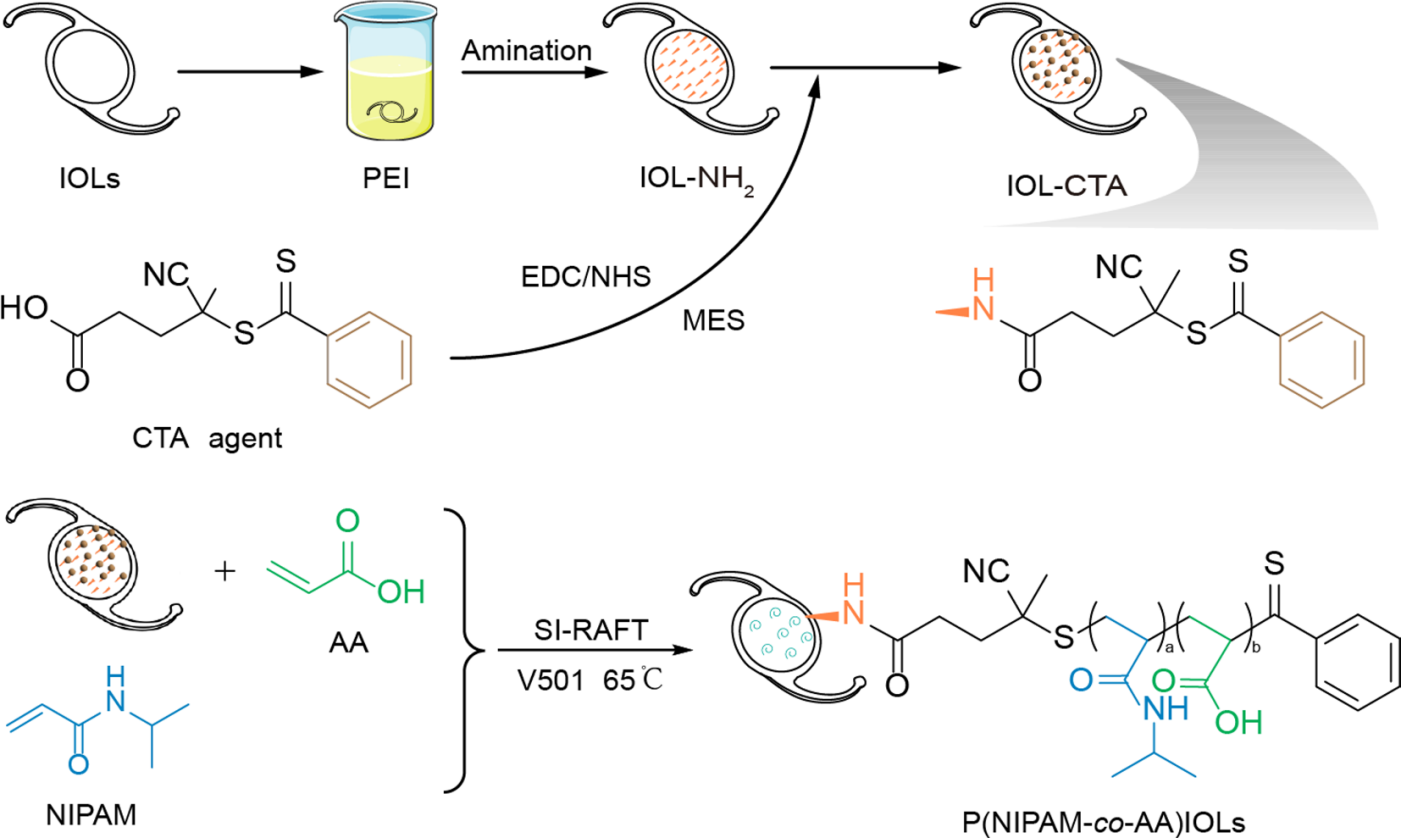
Schematic illustration of the synthesis of the P(NIPAM-*co*-AA)

**Fig. 4:**
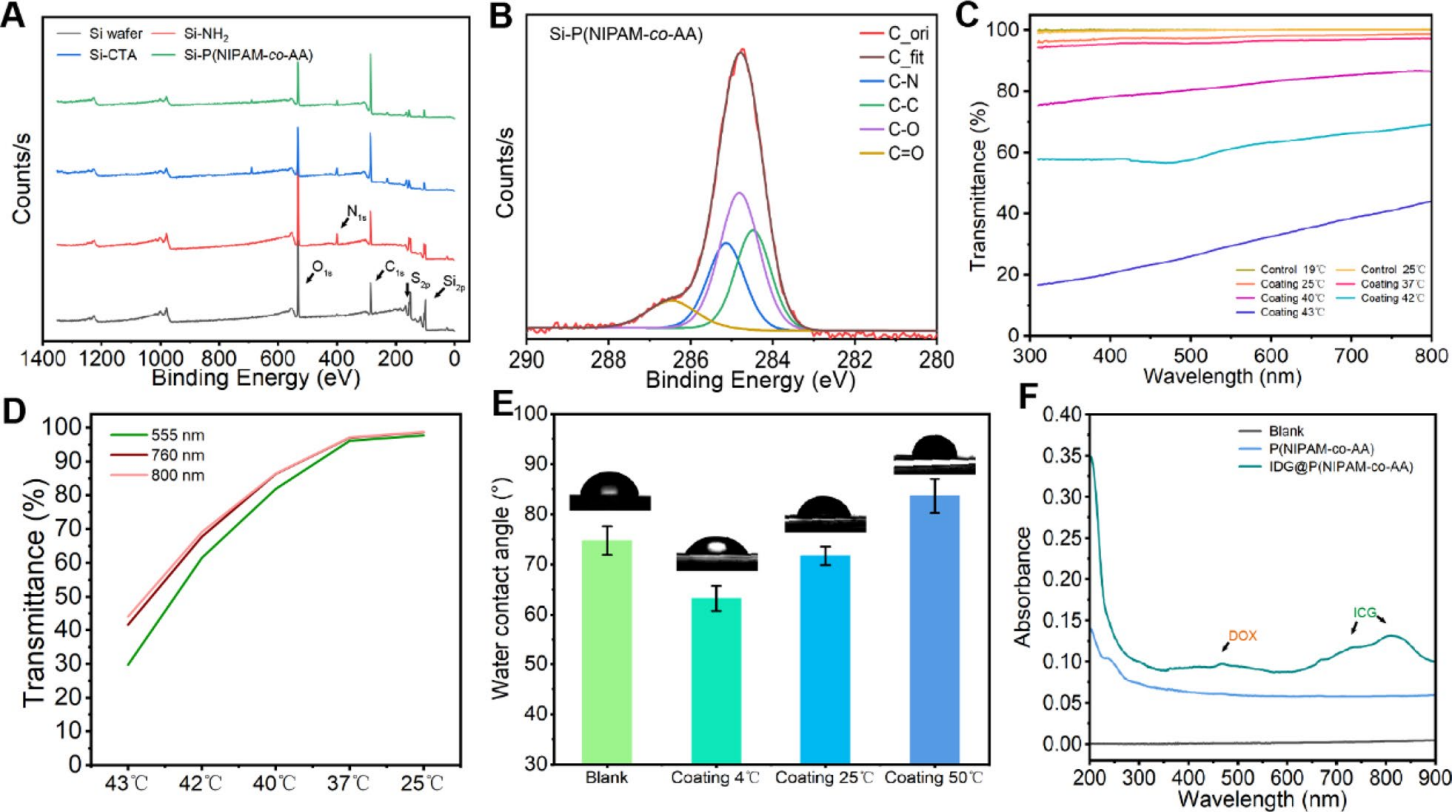
Characterization of P(NIPAM-*co*-AA) brush coating. **A** The XPS curve of blank Silicon wafer, aminated surface Si-NH_2_, Si-CTA and Si-P(NIPAM-*co*-AA). **B** The XPS peak deconvolution and fitting of P(NIPAM-*co*-AA). **C** Transmittance spectrum changes of P(NIPAM-*co*-AA) coating under temperatures from 25 ~ 43 °C. **D** Transmittance spectrum changes of P(NIPAM-*co*-AA) coating at 555, 760 and 800 nm under temperatures from 43 to 25 °C. **E** The water contact angle of blank (25 °C) and P(NIPAM-*co*-AA) brush surface (4, 25 and 50 °C, respectively). **F** UV − vis spectra of pristine material (Blank), P(NIPAM-*co*-AA) brush and IDG@P(NIPAM-*co*-AA)

As shown in Fig. 3, the P(NIPAM-*co*-AA) brush coating was synthesized by SI-RAFT polymerization. To determine the successful synthesis of P(NIPAM-*co*-AA) brush coating, the XPS was measured to analysis the chemical element change of the coating’s surface during the process of IDG@P(NIPAM-*co*-AA) synthesis. The silicon wafer is used as the base material, and the unmodified silicon wafer is composed of carbon (C), silicon (Si) and oxygen elements (O). According to the substrate and chemical reaction formula (Fig. 4A), several elements are mainly detected including: Si, S, C, N, O. As shown in Table 1, the spectrum of aminated surface exhibit obvious N_1s_ peak (from 1.72% to 6.89%) and lower Si_2p_ contents (from 35.58% to 22.29%) compared with untreated silicon wafer, indicating the amino introduction by PEI. Though the condensation reaction between the carboxyl groups and amino groups, the Si_2p_ content of the coating further decreased to 9.23%, and the C_1s_, S_2P_ were observed increased from 36.68% to 57.68%, 2.96% to 4.54% respectively. After SI-RAFT polymerization, the higher C_1s_ contents were as excepted observed increased to 66.83%, due to the CTA agent and polymerized monomer (NIPAM and AA). Also, lower Si_2p_ content was also detected to be much lower than aminated surface (from 9.23 to 7.87%). In addition, the N_1s_ content had no obviously change, this maybe the signal of N_1s_ from NIPAM compensated for the N_1s_ signal on the aminated surface. Furthermore, the C_1s_ peaks of P(NIPAM-*co*-AA) surface changed from 28.74% to 66.83% compared with the unmodified surface, and its peak scan revealed the existence of C–N, C–C, C–O C=C (Fig. 4B). These results indicated that we successfully synthesized the P(NIPAM-*co*-AA) brush coating on the surface through SI-RAFT polymerization.

**Table 1:** The XPS element atomic of Si wafer, Si-NH_2_, Si-CTA, Si-CTA, Si-P(NIPAM-*co*-AA)

Element atomic %					
surface name	Si_2p_	S_2p_	C_1s_	N_1s_	O_1s_
Si wafer	35.58	7.46	28.74	1.72	26.5
Si-NH_2_	22.29	2.96	36.68	6.89	31.19
Si-CTA	9.23	4.54	57.68	6.88	19.53
Si- P(NIPAM-*co*-AA)	7.87	2.56	66.83	3.07	18.58

Afterwards, transmittance analysis of P(NIPAM-*co*-AA) brush coating was also performed by UV–vis. In order to simulate the intraocular environment, the detection was operated by soaking the coating into water. Figure 4C revealed the coating transmittance of P(NIPAM-*co*-AA) brushes at different environment temperatures via changing the temperature of the water. Unmodified quartz flakes were as control. Obviously, the transmittance of unmodified quartz flakes would not change when the temperature changed. However, when the coating temperature (T_C_) was adjusted to 25 and 37 °C, the transmittance was 97.7% and 96.1% at 550 nm (green light, the most sensitive band of the human eye), which exhibit a good light transmittance. However, when the T_C_ was raised to 40 °C, the transmittance was drops to 86.4%, and as the T_C_ continues raised to 42 and 43 °C, the transmittance decreased. Also, similar phenomena could be observed in red and NIR bands. These phenomena revealed that the P(NIPAM-*co*-AA) brush coating has the characteristic of temperature response, and its T_LCST_ was about 40 °C. Optical transmittance of the coating remained practically unchanged when the T_C_ < T_LCST_, while the transmittance decreased when the T_C_ > T_LCST_. Furthermore, due to the role of NIPAM, the change in transmittance with temperature was reversible, when the temperature dropped from 43 to 25 °C, the transmittance will return to the previous state (Fig. 4D). Hence, optical transmittance characterizations evidence the successful formation of P(NIPAM-*co*-AA) brush coating and suggested that this coating exhibited obvious temperature-responsive properties when T_C_ < T_LCST_ or T_C_ > T_LCST_.

Then, the P(NIPAM-*co*-AA) brush surface was then characterized with WAC measurements at 4, 25 and 50 °C. As shown in Fig. 4E, the WAC of blank surface was 74.7 ± 2.8° and P(NIPAM-*co*-AA) coating was 71.7 ± 1.8° at 25 °C. Apparently, P(NIPAM-*co*-AA) brush surface behaved more hydrophilic than the blank one. This is due to the amide group on the P(NIPAM-*co*-AA) chain interacts with water molecule to form a strong hydrogen bond to stretch the molecular chain and then present a hydrophilic surface [42]. In addition, the WAC of P(NIPAM-*co*-AA) increased to 83.6 ± 3.4° with increasing temperature (from 25 to 50 °C). This is because the hydrogen bond between the amide group and the water molecule in the polymer chain is broken (T_C_ > T_LCST_), the hydrophobic force between the isopropyl groups is strengthened, the entire chain collapses and contracts, and the whole surface develops in the hydrophobic direction [43].

Afterward, the IDG@P(NIPAM-*co*-AA) was prepared by dipping the coating into the IDG NPs solution. Firstly, the P(NIPAM-*co*-AA) brush coating was immersed in hot water at 50 °C, due to the temperature sensitivity of NIPAM, the polymer brushes into a clump and expelled moisture. Subsequently, the coating was soaked in a cold IDG NPs solution at 4 °C, then the polymer brush stretched and IDG NPs was loaded in it. As shown in Fig. 4F, the characteristic absorption peaks of IDG NPs (ICG and DOX) were detected in IDG@P(NIPAM-*co*-AA) brush coating according to UV–vis spectrum.

### Photothermal and photodynamic properties of IDG@P(NIPAM-*co*-AA)

Since IDG@P(NIPAM-*co*-AA) brush coating was designed to take advantage of its temperature-sensitive properties to control the drug release and its photo-chemical therapy, we used IR thermal camera and UV–vis to monitor vitro photothermal and photodynamic performance with NIR irradiation.

**Fig. 5:**
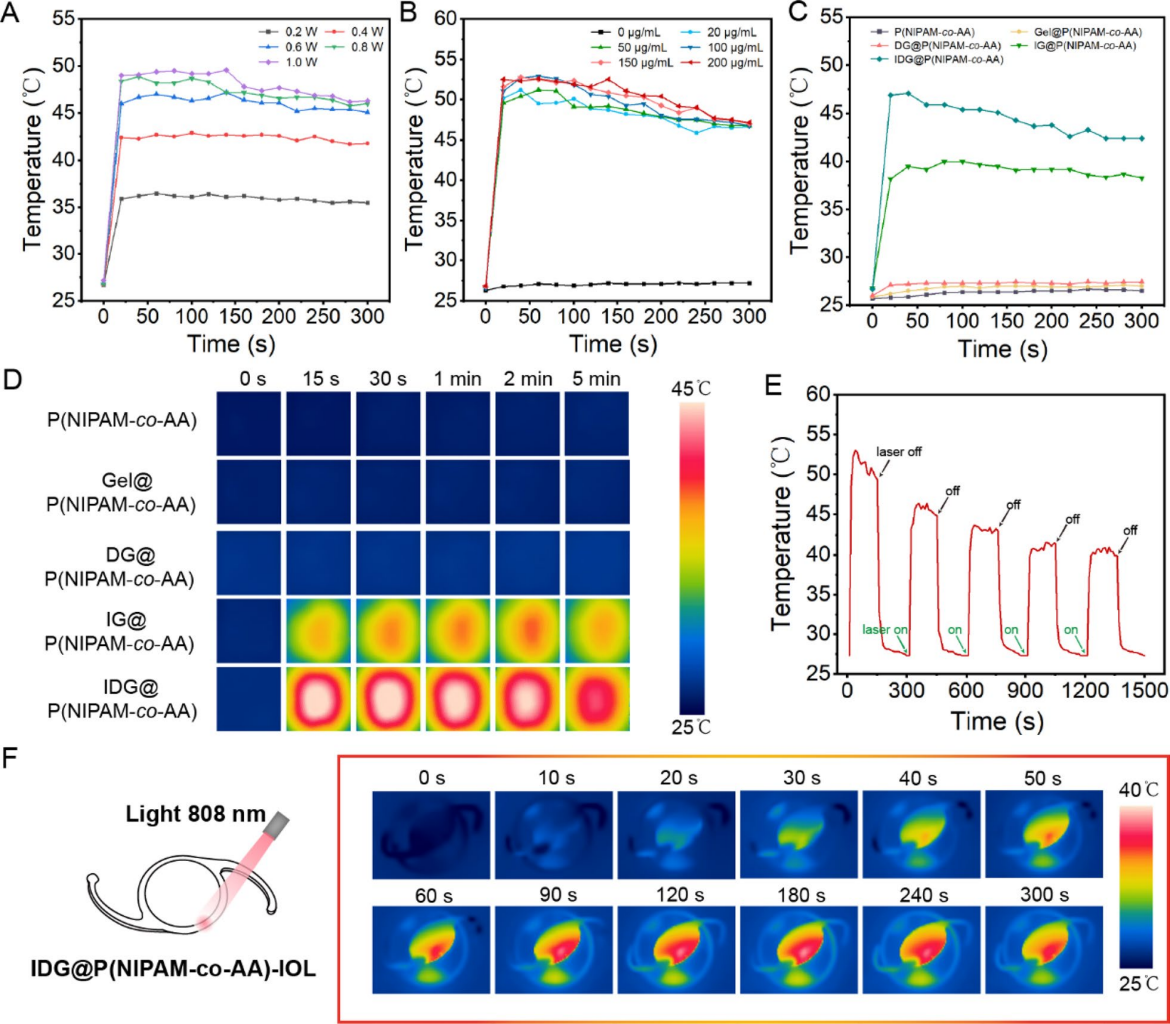
Photothermal performance of IDG@P(NIPAM-co-AA) brush coating. **A** ~ **B** Power dependent photothermal curve of IDG@P(NIPAM-*co*-AA) surface (immersion concentration: 50 µg/mL) and different immersion concentration operated photothermal curve of IDG@P(NIPAM-*co*-AA) surface (laser power: 1.0 W/cm^2^). **C** In vitro PTT profiling of different surfaces as mentioned at immersion concentration of 100 µg/mL, following 808 nm laser irradiation (0.38 W/cm^2^). **D** Thermal images of different coating surfaces. **E** Photothermal stability of IDG@P(NIPAM-*co*-AA) for successive five cycles of on/off laser irradiation. **F** Photothermal range of IDG@P(NIPAM-*co*-AA)-IOLs with a laser irradiation deliberately on the edge of the lens

Under different immersion concentrations or laser power densities, IDG@P(NIPAM-*co*-AA) brush coating displayed remarkable photothermal property. As shown in Fig. 5A, when the initial IDG NPs immersion concentration was 50 µg/mL, a maximum temperature increase was recorded as 49.6 °C (the laser-power 1.0 W/cm^2^). Two major temperature increases could be observed when power was increased from 0.2 to 0.4 W/cm^2^ and from 0.4 to 0.6 W/cm^2^, while this phenomenon was not observed in subsequent power boosts. In addition, the temperature of coating was observed to rise first and then fall a little, especially in power 1.0, 0.8, and 0.6 W/cm^2^. However, under the low-power laser irradiation (0.2 and 0.4 W/cm^2^), the coating remained a relatively stable temperature. This result may be due to the fact that lower power could effectively avoid rapid consumption of photosensitizers in the coating during a short period of NIR irradiation.

After that, we examined the relationship between immersing concentration and temperature rise (laser- irradiation power fixed at 1.0 W/cm^2^), we found that when the immersing concentration was between 20 and 100 μg/mL, the maximum temperature was positively correlated with immersion concentration. However, when the concentration exceeded 100 μg/mL, the temperature change was not obvious (Fig. 5B).

All above results suggested that the increase of laser-power indeed affected the magnitude of the temperature increase, but was also limited by the number of IDG NPs that loaded on the coating. Meanwhile, the immersing concentration could change the temperature rise range to some extent, but when the loaded nanoparticle reached to coating’s the upper limit, the effect of increasing the immersing concentration was not good. To guarantee the therapeutic effect of local photothermal therapy, we need to keep the temperature rise up to at least 45 °C [44]. At the same time, taking into account the needs of coating temperature behavior (T_C_ > T_LCST_), we finally chose the laser-power 0.38 W/cm^2^ and immersion concentration 100 μg/mL.

Under the two conditions mentioned above, the photothermal properties of the coating were examined again through real-time temperature monitoring. As a results, the temperature increase of the P(NIPAM-*co*-AA), Gel@P(NIPAM-*co*-AA), DG@P(NIPAM-*co*-AA), IG@P(NIPAM-*co*-AA) and IDG@P(NIPAM-*co*-AA) were recorded as 1 °C, 1.2 °C, 1.4 °C, 13.2 °C and 17 °C, respectively (Fig. 5C). The temperature of P(NIPAM-*co*-AA), Gel@P(NIPAM-*co*-AA) and DG@P(NIPAM-*co*-AA) was merely increases about 1 °C. Notably, the IG@P(NIPAM-*co*-AA) and IDG@P(NIPAM-*co*-AA) coating temperature peaked at 50 s and remained stable thereafter under 0.38 W/cm^2^ for 5 min. Thermal images in Fig. 5D obviously showed the surface temperature of different coating groups during heating process. The thermal images of coating IDG@P(NIPAM-*co*-AA) suggested that it had good photothermal properties and could reach temperature peaks in a short time. Besides, we noticed that the area with the highest temperature was almost the same size of the light spot, and only slightly temperature increase in the area outside the light spot. This is meaningful to reduce the unintentional damage of surrounding health tissue. As expected, we found the coating without IG or IDG NPs loading would not be heated under NIR irradiation. This also proved from the side that the existence of ICG was the key to the heat generation of the coating.

As the stability of the IDG@P(NIPAM-*co*-AA) brush coating was highly desirable to make sure high PTT efficacy, we determined the photothermal stability under five on/off laser cycles (Fig. 5E). During five consecutive laser on/off cycles, IDG@P(NIPAM-*co*-AA) always reached maximum temperature within 50 s and maintain a relatively stable temperature. When laser is off, the temperature of the IDG@P(NIPAM-*co*-AA) drops rapidly to the initial level. There was no obvious change detected in the photothermal efficacy during the five cycles. The above results demonstrated that IDG@P(NIPAM-*co*-AA) had excellent stability and could be excited by intermittent NIR. In addition, we found that the maximum temperature decreased after each laser on/off cycle, which was due to the consumption of IDG NPs.

Finally, we observed the range of temperature increase under laser irradiation by deliberately deflecting the direction of light to the edge of the IOLs (Fig. 5F). As a result, even irradiated at the edge of IDG@P(NIPAM-*co*-AA) IOL, the temperature of whole IOL was still raised, and the temperature of contralateral edge was raised at least 8 °C. This phenomenon suggested that photothermal range was limited and the heat conduction behavior of the coating was enough to reach T_LCST_ even the edge, considering the temperature of eye is just a little lower than the human body.

### Photothermal drug release behavior

**Fig. 6:**
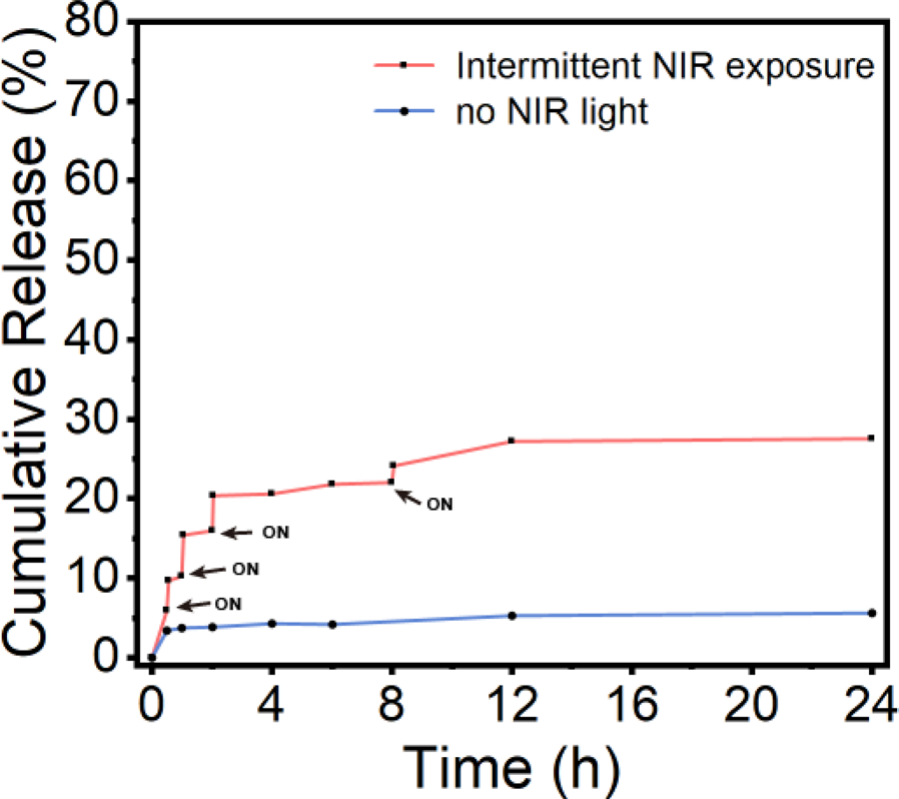
Cumulative release profiles of IDG NPs from IDG@P(NIPAM-*co*-AA) with or without laser irradiation

To confirm our photothermal release concept, we further investigated the release behavior of IDG NPs from IDG@P(NIPAM-*co*-AA) under laser irradiation and without laser irradiation (as a control). Impressively, more IDG NPs released from the nanocomposite under laser irradiation compared to the control group (Fig. 6). In detail, merely a little IDG NPs released from IDG@P(NIPAM-*co*-AA) was detected without irradiation, which suggested that the stretched P(NIPAM-*co*-AA) brush can well prevent the leakage of IDG NPs. However, once we gave it NIR irradiation, the coating was heated and showed a rapid release behavior of IDG NPs, which occurred in all 4 light-on cycles. Based on these findings, we concluded that IDG@P(NIPAM-*co*-AA) had notable photothermal release behavior in connection with the P(NIPAM-*co*-AA) brush coating and IDG NPs heating was exists. Then, 2% SDS solution was used to elute the coating for loaded drug content. As a results, the whole coating had a drug loading of approximately 28 µg (25 µg/cm^2^).

### Photodynamic behavior of IDG@P(NIPAM-*co*-AA)-IOL

**Fig. 7:**
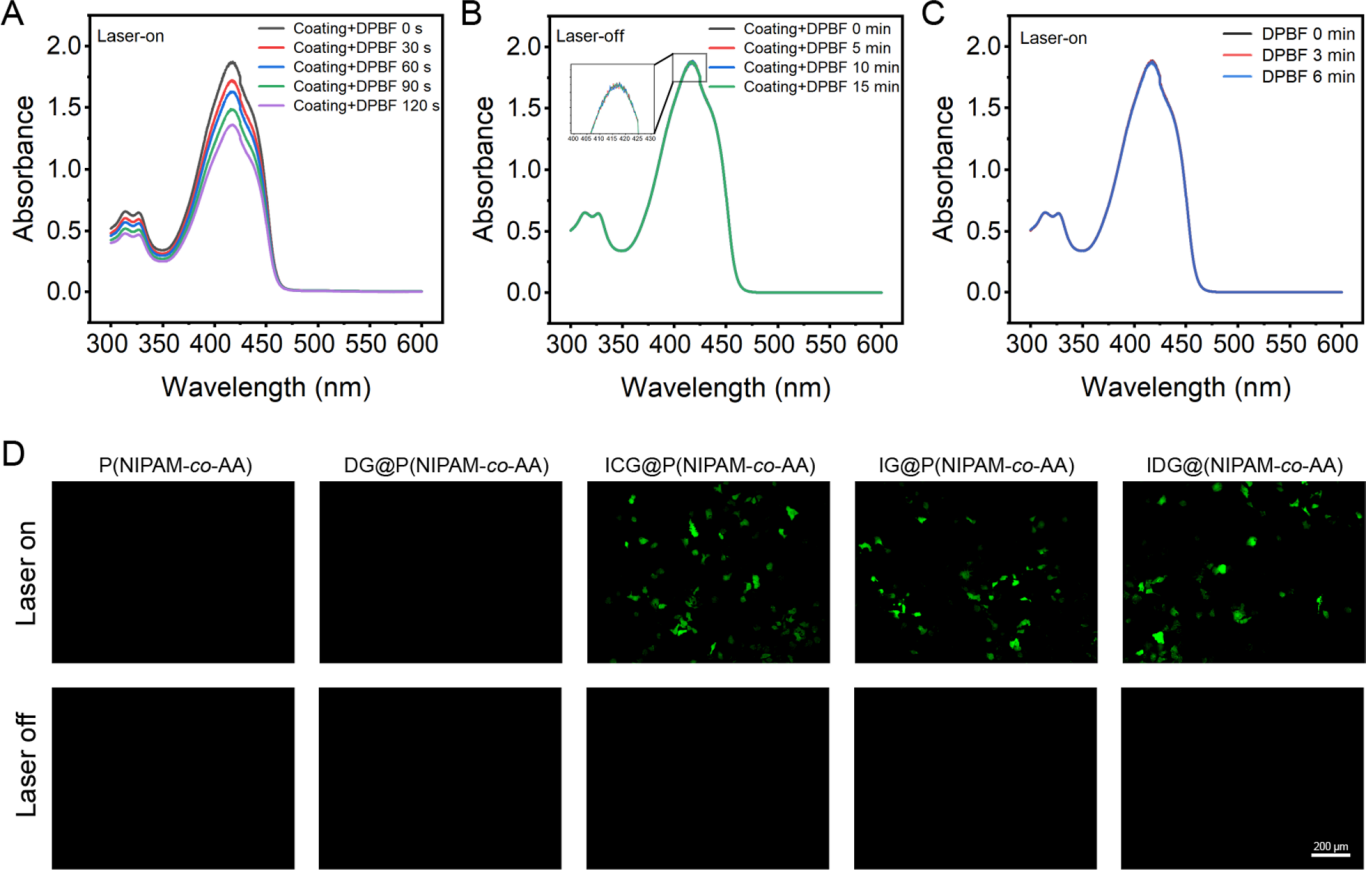
Photodynamic behavior of IDG@P(NIPAM-*co*-AA)-IOL. **A**, **B** Time dependent decrease of DPBF treated IDG@P(NIPAM-*co*-AA)-IOL via UV–vis with or without NIR irradiation. **C** Absorbance changes of DPBF alone followed by 808 nm laser irradiation (0.38 W/cm.^2^) as a control. **D** Fluorescence images of HLECs incubated with DCFH on different P(NIPAM-*co*-AA) coatings’ surface with or without NIR irradiation (Scale bars = 200 µm)

In order to verify simultaneous PDT by thermally released from IDG@P(NIPAM-*co*-AA)-IOL under NIR irradiation, ^1^O_2_ generation was determined by DPBF, a well-known ^1^O_2_ indicator. The generation of ^1^O_2_ was determined by a gradual decay of the DPBF absorption peak at 410 nm. As shown in Fig. 7A, the IDG@P(NIPAM-*co*-AA)-IOL under 808 nm irradiation (0.38 W/cm^2^) exhibited noticeable time dependent decrease in the DPBF absorption peak at 410 nm, suggesting ^1^O_2_ efficient generation. In contrast, the absorption peak of DPBF remain unchanged in the light-off situation (Fig. 7B), which confirmed that the merely IDG@P(NIPAM-*co*-AA)-IOL did not generate ^1^O_2_. As a control, DPBF alone showed no photodegradation ability under NIR irradiation, which confirming the photostability of DPBF (Fig. 7C). The above results proved the ^1^O_2_ generation ability of IDG@P(NIPAM-*co*-AA)-IOL with NIR irradiation.

Encouraged by the excellent intracellular ^1^O_2_ generation of IDG@P(NIPAM-*co*-AA)-IOL detected by the DPBF, we then studied the vivo ^1^O_2_ generation efficiency of IDG@P(NIPAM-*co*-AA) synthesized at pristine material sheet. In contrast, DG@P(NIPAM-*co*-AA), ICG@P(NIPAM-*co*-AA) and IG@P(NIPAM-*co*-AA) coating were also prepared with similar method. The P(NIPAM-*co*-AA) alone was as a control. Then, intracellular ^1^O_2_ generation was detected by monitoring the green fluorescence from the oxidized DCF. As shown in Fig. 7D, ICG@P(NIPAM-*co*-AA), IG@P(NIPAM-*co*-AA) and IDG@P(NIPAM-*co*-AA) coating all exhibit obvious DCFH signals under irradiation resulted from the intracellular ^1^O_2_ generation. While this phenomenon did not appear in other groups. At the same time, no green fluorescence signal was detected without NIR irradiation. These results were well consistent with previous DPBF photodegradation, which confirmed that IDG@P(NIPAM-*co*-AA) could be used for PDT therapy.

### Optical properties of IDG@P(NIPAM-*co*-AA)-IOL

**Fig. 8:**
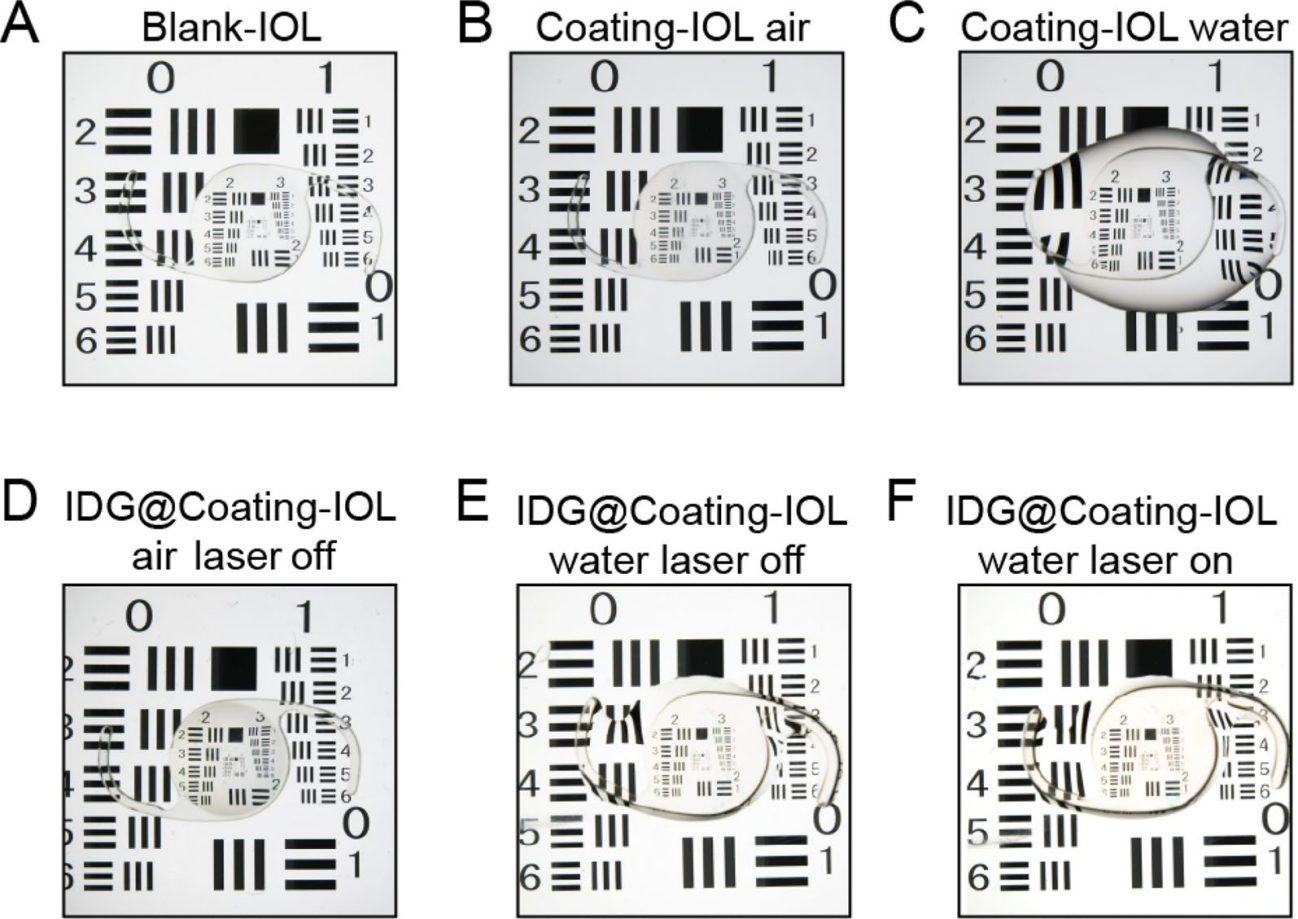
Optical properties of different IOLs including Blank-IOL (**A**), P(NIPAM-*co*-AA)-IOL in air at 25 °C (**B**, Coating-IOL, air), P(NIPAM-*co*-AA)-IOL in water (**C**, Coating-IOL, water); IDG@P(NIPAM-*co*-AA)-IOL in air (D, IDG@Coating-IOL, air), IDG@P(NIPAM-*co*-AA)-IOL in water (IDG@Coating-IOL water) without or with NIR irradiation (**E**, **F**)

As the IOL is an optical element, its transparency is a particularly important property. The resolution plate was used to detect the change of optical properties before and after coating modification. As shown in Fig. 8, through Blank-IOL, P(NIPAM-*co*-AA)-IOL (Coating-IOL) and IDG@P(NIPAM-*co*-AA)-IOL (IDG@Coating-IOL), all lines and numbers of the resolution could be clearly observed. While, the transparency of the Coating-IOL and IDG@Coating-IOL was a slight lower than that of the Blank-IOL when in air condition (Fig. 8A, B and D). However, the transparency of Coating-IOL and became better after it was covered with a little water (Fig. 8B and D). This was due to the role of hydrogen bonds between NIPAM and water, the polymer brush is stretching when T_C_ was below the T_LCST_. The same phenomenon also appeared in IDG@Coating-IOL(Fig. 8D and E). What’s more, it became redder after NIR irradiation for 5 min according to Fig. 8E and F. This result suggested that some IDG nanoparticles were released after NIR irradiation, which also proved the photothermal release behavior.

### Synergistic inhibition effect of drug-phototherapy

**Fig. 9:**
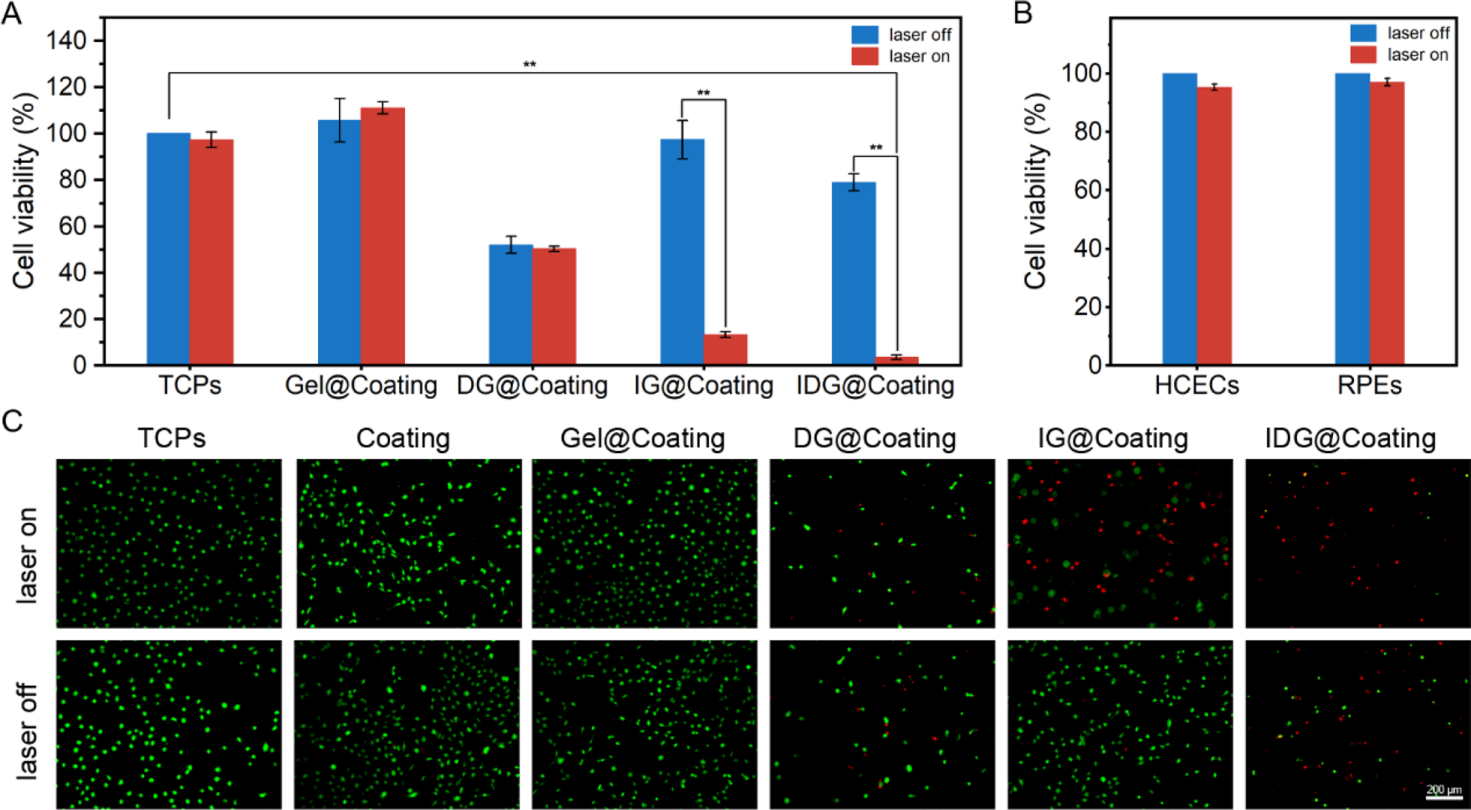
**A** Viability of HLECs on TCPs, Gel@Coating, DG@ Coating, IG@P Coating and IDG@ Coating without or with NIR irradiation. **B** Viability of HCECs and RPEs without or with 0.38 W/cm^2^ NIR irradiation. **C** Fluorescence images of live/dead staining of HLECs on different coatings with or without laser treatment for 5 min. The live cells were stained with AM (green) and the dead cells were stained with PI (red). Scale bars = 200 µm

The antiproliferative effects of dark/light cytotoxicity on HLCEs were investigated with a CCk-8 analysis. HLECs seeded on the tissue culture polystyrene (TCPS) was operated follow the same treatment as a control. As shown in Fig. 9A, under no laser irradiation, the cell viability of Gel@P(NIPAM-*co*-AA) and IG@P(NIPAM-*co*-AA) coating was both over 95%, which means P(NIPAM-*co*-AA), Gel NPs and ICG did not exhibit significant cytotoxicity. Then we found a decrease of cell viability in DG@P(NIPAM-*co*-AA) and IDG@P(NIPAM-*co*-AA) coating, this might due to the cellular endocytosis of some nanoparticles that contained DOX. Meanwhile, obvious light and dark toxicity changes were observed in IG@P(NIPAM-*co*-AA) and IDG@P(NIPAM-*co*-AA). After laser irradiation, the viability of HLECs decreased to about 13.2% in IG@P(NIPAM-*co*-AA), which revealed that the behavior of encapsulated nanoparticles and loaded into coating did not affect the therapeutic effect of ICG. Similarly, we found about 30% HLECs on IDG@P(NIPAM-*co*-AA) were killed due to the cellular endocytosis of IDG NPs when laser off. And when laser on, more than 95% cells were dead which was attributed to the combined effect of phototherapy and drug-therapy (ICG-mediated PTT&PDT and the rapid IDG NPs release triggered by hyperthermia). Meanwhile, we also evaluated the influence of 0.38 W/cm^2^ NIR laser irradiation for 5 min to HCECs and RPEs. As a results, the above conditions showed no obvious damage to both cells.

The live (green)/dead (red) staining was further used to evaluate the in vitro drug-phototherapy, which was consistent with the CCK-8 results (Fig. 9C). In detail, almost all HLECs incubated with IDG@P(NIPAM-*co*-AA) surface were dead and exhibit the most intensive red fluorescence under NIR irradiation. At the same time, no obvious red fluorescence signals were detected in groups of DG@P(NIPAM-*co*-AA) and IDG@P(NIPAM-*co*-AA).

### In vitro cellular uptake and intracellular distribution

**Fig. 10:**
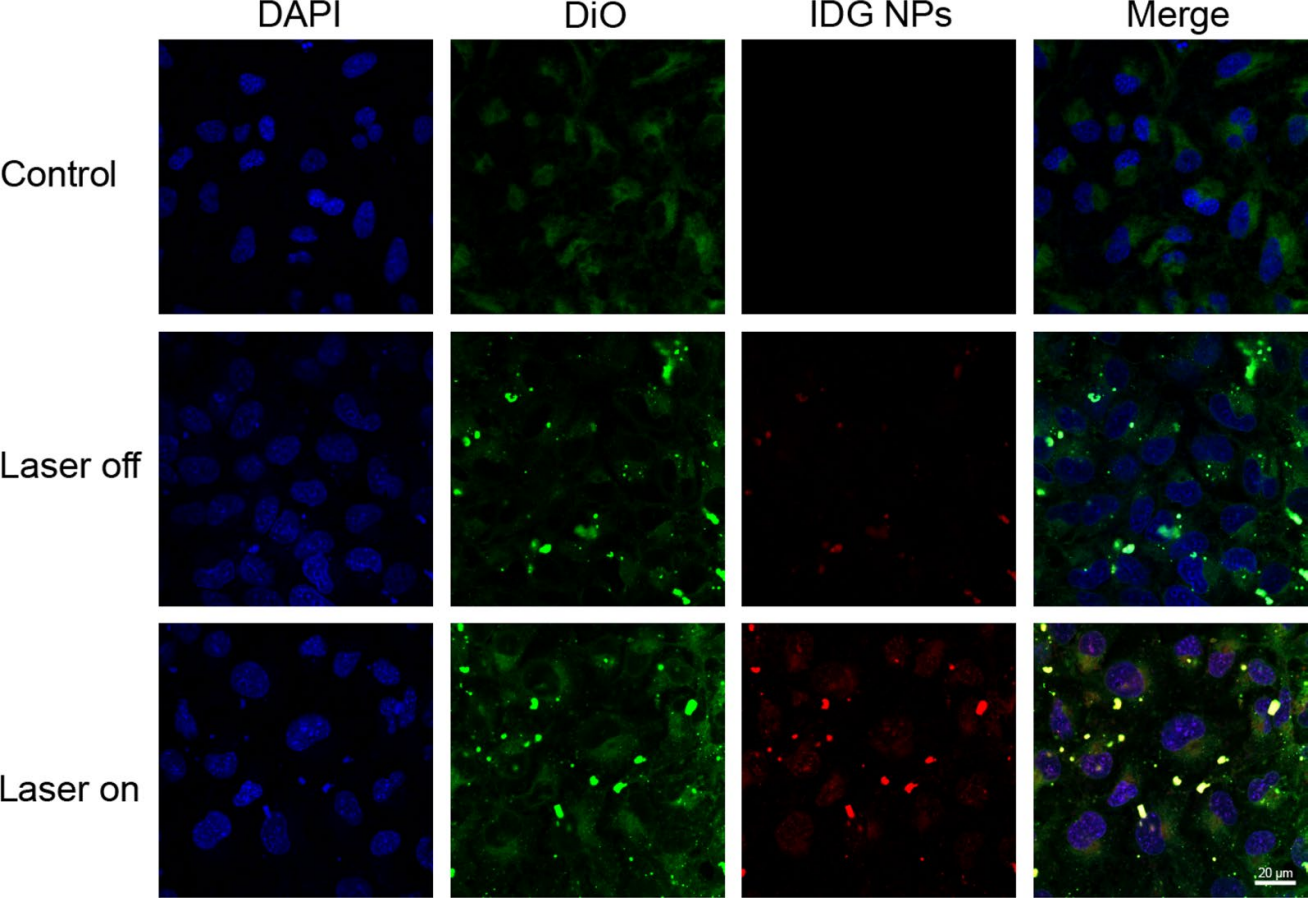
Confocal laser scanning microscopy images of HLECs treated with IDG@P(NIPAM-co-AA) coating after 4 h incubation with or without laser irradiation (red = IDG NPs, green = cells membrane, blue = cell nucleus). Scale bars = 20 µm

For better understand the effect mechanism of IDG@P(NIPAM-*co*-AA) brush coating. The cellular uptake and intracellular distribution analysis was operated by CLMS. The HLECs were firstly co-incubated with the coating for 24 h. Then they were operated without or with laser irradiation for 5 min. Same batch HLECs were planted on clean climbing tablets as a control. As shown in Fig. 10, a little red fluorescence signal of IDG was observed after co-incubation, suggesting that cell might have the behavior of eroding coating. Compared with the laser off group, much more red fluorescence signals appeared after NIR laser irradiation and mainly distributed in the cytoplasm, which also indicated that more IDG NPs appeared in the extracellular environment. These phenomea proved that P(NIPAM-*co*-AA) brush coating could prevent the outflow of IDG NPs to a certain extent when T_C_ below T_LCST_. In addition, when under NIR irradiation, IDG not only performed in suit PTT and PDT, also the produced heat that provided a trigger for controllable drug release.

### In vivo effects of drug-phototherapy on PCO inhibition

**Fig. 11:**
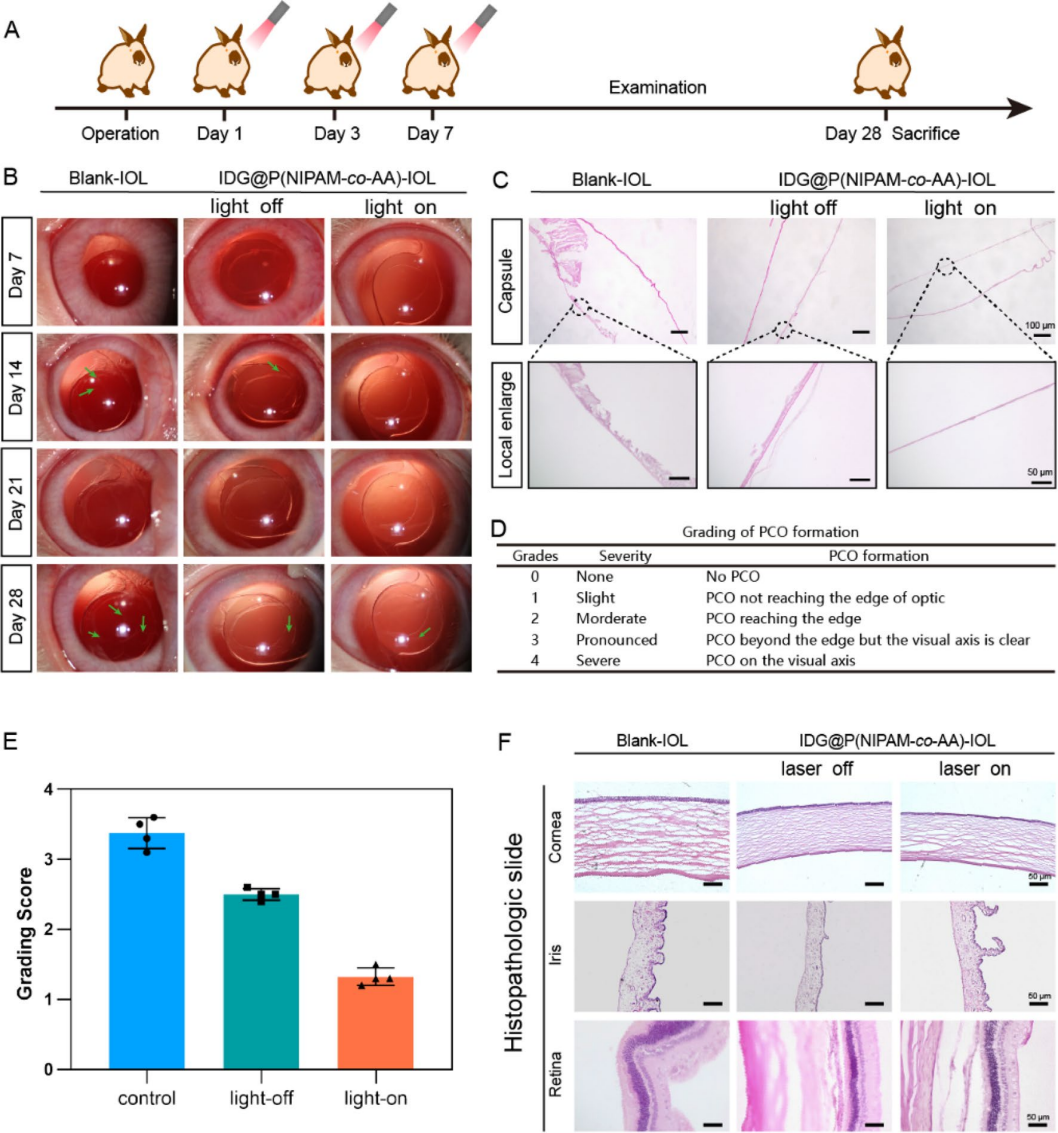
In vivo photo-drug-therapy effect for PCO prevention. **A** Operation time flow of IDG@P(NIPAM-*co*-AA)-IOL implantation after surgery with 808 nm light irradiation; **B** Typical slit lamp microscope images and the local enlarge image of each group at 28 days; **C** Pathological tissue section of capsule from different groups; **D** Grading standards of PCO formation; **E** Grading Score of PCO formation in each group; **F** Pathological results of other tissues of the eye

After confirming the in vitro performance of our IDG@P(NIPAM-*co*-AA), in vivo drug-photo synergistic inhibit effect on PCO was conducted by implanting IDG@P(NIPAM-*co*-AA)-IOL into PCO rabbit eye. As a control, unmodified IOLs (Blank-IOLs) were also implanted. Levofloxacin eye drops and tobramycin eye ointment were used three times a day in the first week to prevent infection and reduce inflammation after surgery. PCO models are typically created by implanting a commercial IOL in the eye of a two-month-old rabbit. Then, LECs accumulated on the surface within two weeks after lens removal due to rapid cell proliferation in the rabbit model [6a]. Then the IDG@P(NIPAM-*co*-AA)-IOLs and Blank-IOLs were treated with NIR irradiation (808 nm, 0.38W/cm^2^) for 5 min at postoperatively 1d, 3d and 7 d (Fig. 11A). The slit lamp was used to observe posterior capsule of rabbits at day 7, 14, 21, 28 (Fig. 11B). Slit-lamp images showed no significant difference between the three groups after surgery (7d). The lens area was translucent and clean and without apparent inflammatory response, which indicating that IDG@P(NIPAM-*co*-AA)-IOLs had no additional inflammatory stimulus whether under NIR irradiation or not. As expected, postoperative residual lens epithelial cells gradually migrate from the peripheral to the central through proliferation in Blank-IOL group at 14 d. Similarly, this phenomenon was also observed in the laser-off group. While in laser-on group of IDG@P(NIPAM-*co*-AA)-IOLs, the capsule and center remained clean. This phenomenon became more apparent after 28 days. Images of control groups and laser-off showed that there was thick fibrous metaplasia of LECs, indicating that PCO formation. Meanwhile, the capsule of laser-on group in IDG@P(NIPAM-*co*-AA)-IOLs remain clean.

To assess the grading of PCO formation, slit lamp images were used in accordance with previous literature [45] (Fig. 11D). Figure 11E illustrated the PCO gradings at 28d. The mean grading for the IDG@P(NIPAM-*co*-AA) light-on group was 1.32 ± 0.13, which was significantly lower than the Blank-IOL control group (3.37 ± 0.23) and IDG@P(NIPAM-*co*-AA) light-off group (2.50 ± 0.08).

The rabbit’s eyes were then taken out for hematoxylin–eosin (H&E) after they were euthanized. The H&E staining images revealed that there was no fibrous LECs in the posterior capsule of IDG@P(NIPAM-*co*-AA)-IOLs laser-on group (Fig. 11C). while, the other groups showed obvious proliferation of LECs, which was consist with the slit lamp evaluation. All above results suggested that IDG@P(NIPAM-*co*-AA)-IOLs implantation and drug-photo synergistic inhibition triggered by photothermal temperature change could effectively kill residue LECs, preventing PCO. On the other hand, images of pathological sections of other tissues in the eye (Fig. 11F, including cornea, iris and retina) had no apparent structural and morphological abnormalities, which means our IDG@P(NIPAM-*co*-AA)-IOLs had a good biocompatibility whether under NIR irradiation.

**Fig. 12:**
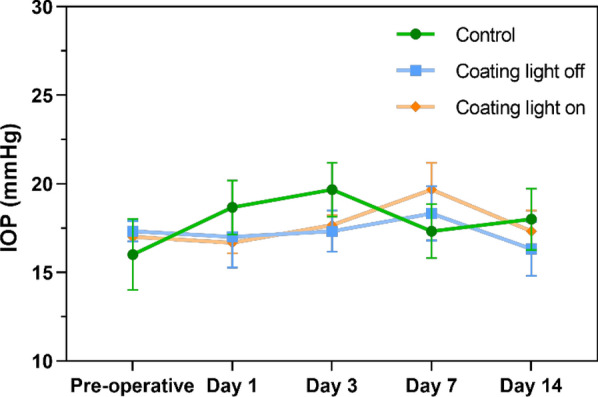
The 14 days IOP changes in control, coating with or without NIR light irradiation

The intraocular pressure (IOP) of animals was also monitored at the determined time (pre-operative, after-operative 1d, 3d, 7d, 14d) after IOL implantation. As shown in Fig. 12, there is no significant difference in IOP changes in the control group or coating group with or without NIR irradiation.

**Fig. 13:**
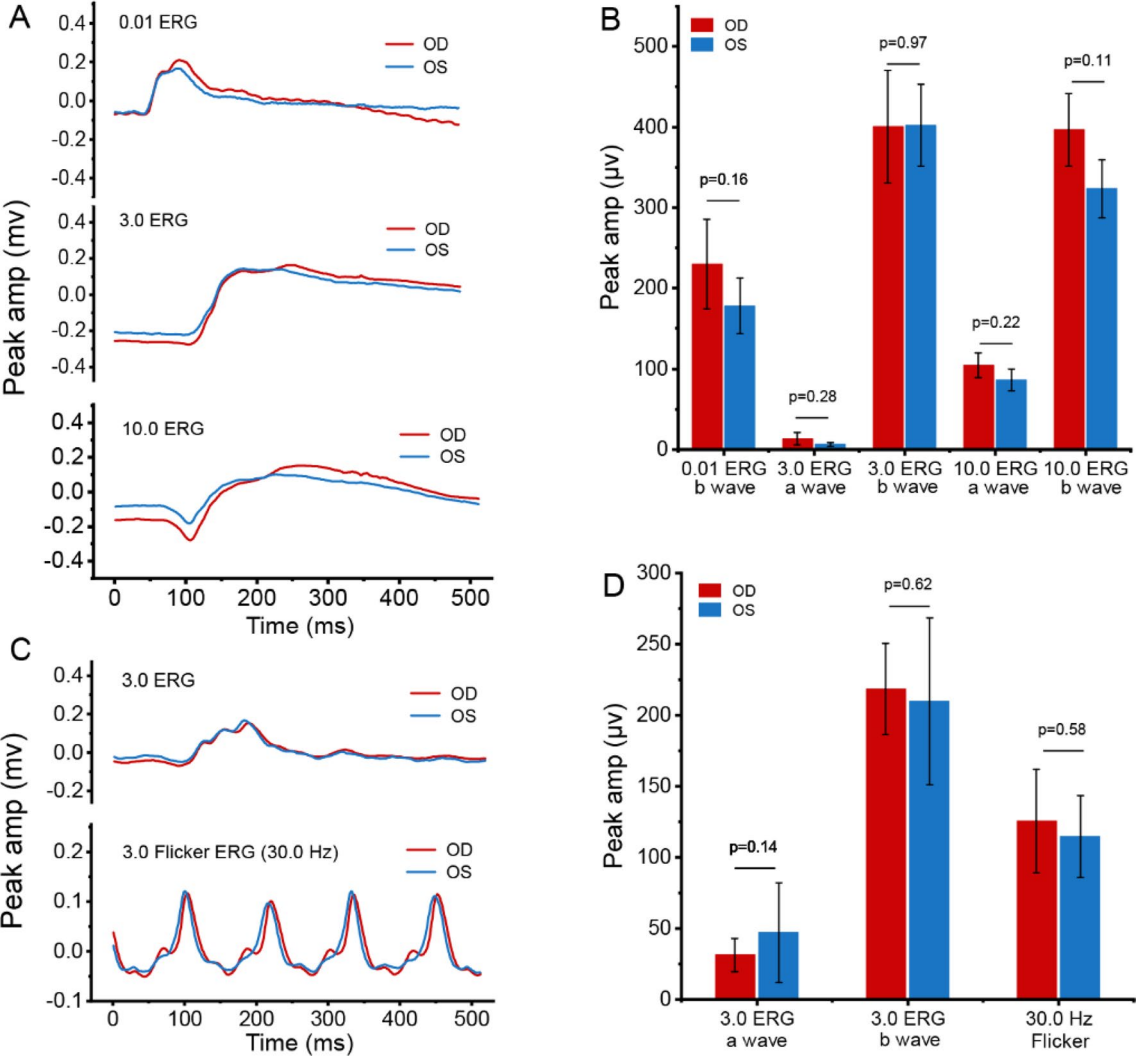
Comparison of ERG data from IDG@P(NIPAM-*co*-AA)-IOL implantation eye with 808 nm laser irradiation eye (OD) and non-surgical eye (OS) of the same rabbit at the 28th day after operation. **A** Dark-adapted flash intensity series of eyes; **B** the quantitative data of dark-adapted peak amplitude; **C** light-adapted flash intensity and flicker response of eyes; **D** the quantitative data of light-adapted peak amplitudes

Furthermore, an electroretinogram exam (ERG) was operated to evaluate the biocompatibility and safety of IDG@P(NIPAM-*co*-AA)-IOLs with 808 nm laser irradiation by functional analysis of retina. Figure 13A exhibited the same individual rabbit’s dark-adapted flash peak amplitude intensity of IDG@P(NIPAM-*co*-AA)-IOL implantation eye and non-surgical eye. In dark-adapted flash program, **b** wave was used to assess the rod response, **a** wave was used to assess photoreceptor-specific function. The a and b wave amplitudes in dark-adapted flash were quantitated in Fig. 13B. As a result, there were no significant differences between the IDG@P(NIPAM-*co*-AA)-IOL implantation eyes and the non-surgical eyes. Also, the light-adapted flash program was operated to evaluate the cone function according to the b wave amplitude (Fig. 13C). The quantitated wave amplitudes result in Fig. 13D showed that the implantation eyes did not significantly differ from the non-surgical eyes even under irradiation. All the above results suggest that the IDG@P(NIPAM-*co*-AA)-IOLs had no functional effect on the retina function, indicating that it has good biocompatibility.

## Conclusion

In this study, we developed a thermosensitive coating for controlled drug-photo therapy that is triggered by low power NIR. The coating offers enhanced security and high efficiency in preventing PCO through the synergistic therapy of phototherapy (PDT&PTT) and drug effect. We utilized IDG NPs to improve the stability of ICG and enable in situ and local PTT&PDT. At the same time, the resulting heat acts as a trigger for controllable drug release from P(NIPAM-*co*-AA). The entire system can be activated with lower-power NIR light. The addition of NIPAM not only facilitates controllable drug release, but also reduces transparency, limiting the effect of light to the IOL surface vicinity. This enhances the safety of light usage. As anticipated, in the animal model of phacoemulsification cataract surgery, IDG@P(NIPAM-co-AA)-IOL demonstrated excellent performance in preventing PCO. The results suggest that the low power NIR triggered thermosensitive controlled drug-photo therapy coating proposed here could have significant potential in the clinical development of PCO prevention. Moreover, this proposed formulation, which utilizes a cascade effect, shows promise as a platform to combine phototherapy and controlled drug-therapy. This could lead to even greater therapeutic potential and improved medication safety.

## Data Availability

All data and materials are shown in the paper and can be directly queried to the corresponding author if further queries are required.
